# Bionic Artificial Neural Networks in Medical Image Analysis

**DOI:** 10.3390/biomimetics8020211

**Published:** 2023-05-22

**Authors:** Shuihua Wang, Huiling Chen, Yudong Zhang

**Affiliations:** 1 School of Computing and Mathematic Sciences, University of Leicester, Leicester LE1 7RH, UK; shuihuawang@ieee.org; 2 Department of Information Systems, Faculty of Computing and Information Technology, King Abdulaziz University, Jeddah 21589, Saudi Arabia; 3 College of Computer Science and Artificial Intelligence, Wenzhou University, Wenzhou 325035, China

Bionic artificial neural networks (BANNs) are a type of artificial neural network (ANN) [1] that draw inspiration from the biological neural networks in living organisms, such as the brain and the nervous system [2]. BANNs aim to create more advanced and adaptable artificial intelligence systems that can learn and make decisions in a way that is more similar to how humans and other animals make decisions [3].

BANNs typically consist of multiple layers of interconnected artificial neurons [4], which process information and make decisions based on input from sensors or other sources. The structure of these networks can vary depending on the task they are designed to perform, but they often include feedback loops and other mechanisms that allow them to learn and adapt over time [5].

There are two advantages of BANNs: (i) First, their ability to learn from experience and adjust their behavior accordingly. This is achieved through a process known as training, in which the network is exposed to a large amount of data and adjusts its weights and connections in response to the patterns it observes [6]. Over time, the network becomes more efficient at processing these data and can make more accurate predictions or decisions. (ii) Second, BANNs can be trained to learn new tasks much faster than traditional ANNs. This is because BANNs can take advantage of the knowledge already stored in the brain [7]. For example, if a BANN is trained to recognize faces, it can then be used to recognize new faces much faster than a traditional ANN that has to learn from scratch.

BANNs have a wide range of potential applications, from computer vision and natural language processing to robotics and control systems. They are particularly well-suited to tasks that require real-time decision-making [8] and adaptability, such as autonomous driving or medical image analysis (MIA) [9].

MIA uses computer algorithms and machine learning techniques to analyze and interpret medical images [10]. MIP roughly contains eight common tasks, as shown in Figure 1. These medical images may be acquired from a variety of imaging modalities, such as X-ray [11], MRI, CT, ultrasound, or microscopy.

Preprocessing improves the quality and relevance of the images by removing noise, enhancing contrast [12], and segmenting the regions of interest. Registration [13] aligns and merges multiple images of the same patient or anatomical region to enable accurate comparisons and analyses. Feature extraction [14] identifies and quantifies relevant characteristics or patterns in the image data to aid in diagnosis or treatment planning.

Classification [15] uses machine learning algorithms to classify or predict a diagnosis or outcome based on the features extracted from the image data. Detection [16] locates and identifies specific structures or abnormalities within an image, such as tumors, lesions, or fractures. Segmentation [17] divides an image into meaningful regions or objects that can be analyzed separately, such as organs, tissues, or lesions. Visualization [18] creates meaningful and informative images or graphical representations of the image data that can aid in diagnosis, treatment planning [19], and communication with patients.

BANNs can help MIA by providing a powerful machine-learning framework [20] that can learn and adapt to the complex and often variable patterns in medical images, enabling more accurate and efficient detection, segmentation, and classification of abnormalities or features of interest.

Additionally, BANNs can incorporate feedback mechanisms [21] and other features that mimic the adaptability and flexibility of biological neural networks, potentially improving the ability of medical image analysis systems to adapt to new and changing situations.

We propose this Special Issue (SI), ‘Bionic Artificial Neural Networks and Artificial Intelligence’, which focuses on the application of BANNs and artificial intelligence (AI) [22] in various fields. The keywords of this SI include the following:

- Bionic artificial intelligence;
- Bionic artificial neural networks;
- Bionic engineering;
- Deep learning;
- Evolutionary machine learning;
- Global optimization;
- Image processing;
- Machine learning;
- Metaheuristic;
- Optimization;
- Supervised learning;
- Transfer learning;
- Transformer;
- Unsupervised learning.

This SI calls for papers that discuss the use of bionic neural networks and AI in areas such as image processing [23], speech recognition, robotics, and control systems. We hope the papers in this SI highlight the benefits of using BANNs, which are designed to mimic the structure and function of the brain [24], in solving complex problems.

## Figures and Tables

**Figure 1:**
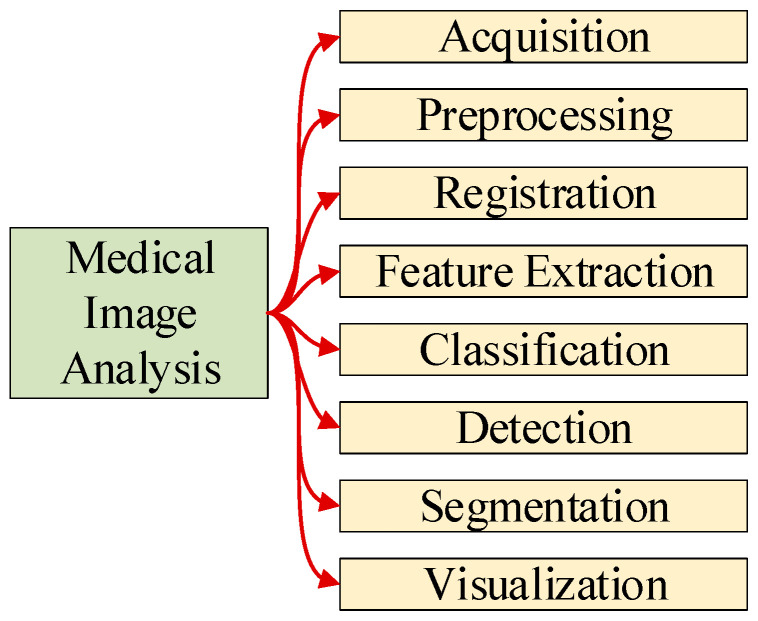
Common tasks in medical image analysis.
